# The Influence of Occupational Noise Exposure on Cardiovascular and Hearing Conditions among Industrial Workers

**DOI:** 10.1038/s41598-019-47901-2

**Published:** 2019-08-08

**Authors:** Xiuting Li, Qiu Dong, Boshen Wang, Haiyan Song, Shizhi Wang, Baoli Zhu

**Affiliations:** 1 Nanjing Prevention and Treatment Center for Occupational Diseases, Nanjing, 210042 China; 2 School of Public Health, South East University, Nanjing, 210000 China; 3 Jiangsu Provincial Center for Disease Control and Prevention, Nanjing, 210028 China

**Keywords:** Occupational health, Epidemiology

## Abstract

This study was conducted to estimate the current prevalence of hypertension, cardiovascular condition and hearing difficulty of workers exposure to occupational noise, and to analyze any associations between these abnormal signs and occupational noise exposure. The subjects included 5205 noise-exposed workers. Workers with high noise exposure were more likely to have a higher threshold value than low exposure ones (*P* < 0.05). Subjects in the high exposure group had a significantly higher risk of hypertension and hearing loss than the ones in low exposure group. Between the ages of 30 and 45, high-level occupational noise exposure led to a significantly raising risk of both hypertension (*Adjusted OR* = 1.59, *95% CI*, 1.19–2.11) and hearing loss (*Adjusted OR* = 1.28, *95% CI*, 1.03–1.60) when comparing to low-level noise exposure. In male workers, the prevalence of hearing difficulty in high exposure group was approximately 1.2 times worse than in low group (*P* = 0.006). In addition, exposure to high noise level demonstrated a significant association with hypertension and hearing loss when the duration time to occupational noise was longer than 10 years. Hypertension and hearing difficulty is more prevalent in the noise-exposed group (higher than 85 dB[A]). Steps to reduce workplace noise levels and to improve workplace-based health are thus urgently needed.

## Introduction

Exposure to occupational noise in relation to occupational injuries has become a legitimate public health issue in recent years. In addition to incurring adverse effects on the auditory system, noise, as a stressor and one of the most harmful agents for workers, may also cause elevated blood pressure, anxiety, angiocardiopathy, and impaired hormone secretion^1–4^. Many studies suggest that along with hearing difficulty, noise exposure has been especially associated with cardiovascular diseases such as arteriosclerosis, hypertension, and coronary heart disease^5–8^. The pathway from noise exposure to these clinical features has been thought to cascade through the autonomic nervous system and endocrine system by a stress response which motivates some biological risk factors for cardiovascular disease (for instance blood pressure and serum lipids)^9^. Despite the above findings, work by other researchers could not find any association between noise exposure and health effects; thus, a consensus concerning this issue is still lacking.^10–13^.

In order to observe whether there is in indeed an association between noise exposure and abnormal health effects such as hypertension, cardiovascular condition or hearing loss, we undertook this study and analyzed data from occupational, physical examinations performed in the Nanjing Prevention and Treatment Center for Occupational Diseases from 2016 to 2017.

Using occupational, physical examination data, the purposes of this study were the following: (1) estimate the current prevalence of hypertension, cardiovascular condition, and hearing difficulty with workers who have been exposed themselves to occupational noise for many years; (2) examine any link these outcomes may have to occupational noise exposure.

## Materials and Methods

### Study population and settings

In this cross-sectional study, we initially recruited 6048 workers with occupational exposure to noise who received both a physical test and assessment in the Nanjing Prevention and Treatment Center for Occupational Diseases. To avoid interference and difficulty in the statistical analysis, those who declined to participate, gave incomplete information about occupational exposure to noise, or did not complete the physical examination report, were excluded from the current research. Subsequently, a total of 843 workers were excluded.

In order to examine the association between occupational noise exposure and health condition, we divided the participants into a high exposure group and a low exposure group and selected 85 dB(A) (the specific domestic noise levels in working places) as the cut-off value for the different noise-exposure groups. Subjects whose exposure level of working noise was less than 85 dB(A) were placed into the low-level group, while those whose exposure was higher than 85 dB(A) were placed into the high-level group. The subjects in high exposure group were selected without any restriction in age or gender. Those in low-exposure group corresponded to subjects in high-exposure group via frequency-matching (1:4) which considered factors of age, exposure time, and sex, and selected individuals from the same company who were having medical examination from the same hospital at the same time. Finally, the study group comprised 5205 noise-exposed workers (1041 high exposure and 4174 low exposure) from factories in Nanjing city.

### Health assessment

Industrial hygienist used a questionnaire for each subject to collected information by face-to-face interviews. The questionnaire obtained personal information including age, length of working years, and history of occupational noise exposure. Health status was evaluated in all subjects by clinical and laboratory examination, which was performed after a face-to-face interview. In their assessment, these health and physical examinations used signs, pulse, blood pressure (BP), electrocardiogram, Ultrasonic B, blood routine, and hepatic function tests.

### Audiometry and noise exposure assessment

According to the Chinese national criteria for measuring noise exposure in the real workplace (GBZ/T 189.8–2007), a sound pressure individual audiometer should be used to evaluate the real working environment. In our study, noise exposure levels were assessed with a sound pressure individual audiometer (Noise-Pro, Quest, USA) at regular time in the day at selected workplaces for three consecutive days twice a year. In order to evaluate the actual noise exposure level, the results were converted to equivalent continuous A-weighted sound pressure from a nominal eight-hours working day (Lex.8 h).

Tone audiometry was conducted by use of the Beckesy audiometer. All subjects underwent a tonal audiometric examination that was performed by an experienced physician. Hearing tests were operated after a period of at least fourteen hours without noise exposure. On the basis of diagnostic criteria (GB 49–2014), both left and right ears were tested by a method of ascending pure tones at frequencies of 500, 1000, 2000, 3000, 4000 and 6000 Hz. Both ears’ high-frequency threshold in average (BHFTA) was calculated as follows:

$${\rm{BHFTA}}=\frac{{{\rm{HL}}}_{{\rm{L}}}+{{\rm{HL}}}_{{\rm{R}}}}{6}$$

BHFTA: Both ears’ high-frequency threshold on average; unit is dB.

HL_L_: Sum threshold of 3000 Hz, 4000 Hz and 6000 Hz of left ear; unit is dB.

HL_R_: Sum threshold of 3000 Hz, 4000 Hz and 6000 Hz of right ear; unit is dB.

### Outcome variables

Blood pressure (BP) was measured according to the standardized WHO method (World Health Organization, 1983). After resting for at least 15 minutes, the subjects were placed in the sitting position during blood pressure measurement. In each subject, two measurements were made and the mean results of the two measurements have been used to represent individuals’ final blood pressure in our study. If the two test results differed by more than 4 mmHg in either systolic blood pressure (SBP) or diastolic blood pressure (DBP), measurements were repeated after a further 10-minute rest until the difference conformed to this criterion. Subjects were diagnosed as hypertension if they reported a previous medical diagnosis of hypertension, or if their mean SBP was higher than 140 mmHg, or if their mean DBP was higher than 90 mmHg.

The electrocardiogram (ECG) results having no disorder according to relevant doctors or health professionals were considered “normal,” and all the others were defined as “abnormal.”

Workers whose BHFTA worse than 25 dB were defined as high-frequency hearing loss, while those less than 25 dB were defined as normal.

### Ethical considerations

This project has been approved by the Ethics Committee of Nanjing Prevention and Treatment Center for Occupational Diseases. All of the participants received a clear explanation of study purposes and procedures and signed informed consents. Ethical considerations have been respected throughout the entire study period.

### Statistical analysis

We used a computerized database (SAS 9.1.3) to analyze all of the data. Continuous data were evaluated using univariate analysis of variance and Student’s t-tests, and qualitative data were analyzed by Pearson χ^2^ contingency tables. We used multivariate logistic regression analysis, calculating crude and adjusted odds ratios (*ORs*) and 95% confidence intervals (*CI*s), to test the level of association between noise exposure and health conditions by controlling potential confounding factors that included age, gender, exposure time to occupational noise and so on. The significance level was set at 0.05.

## Results

### General characteristics of the subjects

Table 1 shows the general characteristics of the subjects who were exposed to occupational noise. There are 5205 subjects who were exposed to workplace noise accepted physical examinations. There were 4164 workers exposed to the low noise level, and 1041 exposed to the high noise level. Of the subjects included, 93.76% were male, and 6.24% were female. The mean age was 37.23 ± 9.11 years, and the mean exposure time to noise was 5.31 ± 5.28 years. There was no significant difference between the low and high noise exposure individuals regarding age, gender and exposure time to noise (Table 1). However, subjects with high noise exposure have a higher threshold value than the low exposure ones (*P* < 0.05).

**Table 1:** Descriptive characteristics of low and high workplace noise exposure subjects.

Variable	All (N = 5205)		High exposure level (N = 1041)		Low exposure level (N = 4164)		*P*
	n	%	n	%	n	%	
Gender							
Male	4880	93.76	976	93.76	3904	93.76	1.000^a^
Female	325	6.24	65	6.24	260	6.24	
Age, years	37.23 ± 9.11		37.06 ± 9.20		37.27 ± 9.09		0.500^b^
**<**30	1292	24.82	263	25.26	1029	24.71	0.502^a^
30–45	2638	50.68	532	51.10	2106	50.58	
**>**45	1275	24.50	246	23.63	1029	24.71	
Exposure time, years	5.31 ± 5.28		5.25 ± 5.17		5.33 ± 5.32		0.680^b^
**<**3	1996	38.35	402	38.62	1594	38.28	0.231^a^
3–10	2325	44.67	484	46.49	1841	44.21	
**>**10	884	16.98	155	14.89	729	17.51	
Threshold [dB (A)]	23.32 ± 10.50		23.53 ± 10.12		22.48 ± 11.87		**0.004** ^b^

^a^Two-sided χ^2^ test for the frequency distributions of selected variables between low and high exposure level.

^b^*t*-test of the difference between the two exposure groups.

### Prevalence of abnormal cardiovascular conditions and hearing difficulty

As can be seen from Table 2, the prevalence of each condition in all participants was as follows: hypertension 11.83%, abnormal ECG 13.20%, and hearing difficulty 22.23%. In the group exposed to a high noise level, the prevalence of hypertension, abnormal ECG, and hearing difficulty was 13.64%, 13.74%, and 25.74% respectively. Meanwhile, in the low noise group, the above prevalence was 11.38%, 13.06% and, 21.35% respectively. We may infer that workers in the high exposure group had significantly higher risks of hypertension and hearing loss than workers who were exposed to a lower noise level; *P* value was 0.047 for hypertension and 0.003 for hearing loss. However, there was no significant difference when concerning the distribution of ECG between the two exposure groups (*P* = 0.573).

**Table 2:** Cardiovascular and hearing conditions of the two exposure groups.

Variable		All (N = 5205)		High exposure level (N = 1041)		Low exposure level (N = 4164)		*P* ^*a*^
		n	%	n	%	n	%	
Hypertension	Without	4589	88.17	899	86.36	3690	88.62	**0.047**
	With	616	11.83	142	13.64	474	11.38	
ECG	Normal	4518	86.80	898	86.26	3620	86.94	0.573
	Abnormal	687	13.20	143	13.74	544	13.06	
Hearing Loss	Without	4048	77.77	773	74.26	3275	78.65	**0.003**
	With	1157	22.23	268	25.74	889	21.35	

^a^Two-sided χ^2^ test for the frequency distributions of selected variables between low and high exposure level.

### Stratified analysis between the two exposure groups by age

We divided age into three stages (**<**30, 30–45, and **>**45). For ages between 30 and 45, a history of high-level occupational noise exposure led to a significantly raising dangerous of both hypertension (*Crude OR* = 1.56, *95% CI*, 1.18–2.07) and hearing loss (*Crude OR* = 1.26, *95% CI*, 1.01–1.58) when comparing to low-level noise exposure, after adjustment for sex and exposure time to occupational noise; the *Adjusted OR* and *95% CI* was 1.59 (1.19–2.11) and 1.28 (1.03–1.60) respectively. However, the difference was not statistically significant when the analysis was limited to those under the age of 30 and over the age of 45 (Table 3).

**Table 3:** Stratified analysis of cardiovascular and hearing conditions between two exposure groups by age.

Variable		High exposure level N (%)	Low exposure level^*c*^ N (%)	*P* value^*a*^	*Crude OR* (*95% CI*)	*Adjusted OR* (*95% CI*)^*b*^
**<30 years**						
Hypertension	Without	254 (96.58)	984 (95.63)	0.605	0.78 (0.37–1.61)	0.78 (0.37–1.61)
	With	9 (3.42)	45 (4.37)			
Hearing Loss	Without	227 (86.31)	927 (90.09)	0.093	1.44 (0.96–2.17)	1.43 (0.95–2.16)
	With	36 (13.69)	102 (9.91)			
**30–45 years**						
Hypertension	Without	456 (85.71)	1903 (90.36)	**0.003**	**1.56** (**1.18–2.07)**	**1.59** (**1.19–2.11)**
	With	76 (14.29)	203 (9.64)			
Hearing Loss	Without	396 (74.44)	1656 (78.63)	**0.041**	**1.26** (**1.01–1.58)**	**1.28** (**1.03–1.60)**
	With	136 (25.56)	450 (21.37)			
**>45 years**						
Hypertension	Without	189 (76.83)	803 (78.04)	0.670	1.07 (0.77–1.49)	1.08 (0.77–1.50)
	With	57 (23.17)	226 (21.96)			
Hearing Loss	Without	150 (60.98)	692 (67.25)	0.072	1.31 (0.99–1.75)	1.32 (0.98–1.77)
	With	96 (39.02)	337 (32.75)			

^a^Two-sided χ^2^ test for comparing the distribution of hypertension, abnormal ECG and hearing loss between two groups.

^b^Adjusted for sex and occupational exposure time for noise in the logistic regression model.

^c^Reference group.

### Stratified analysis between the two exposure groups by gender

**Figure 1:**
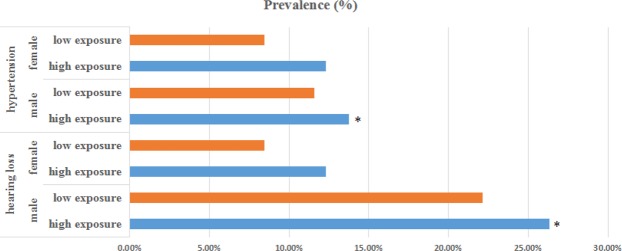
Bar charts for the prevalence of both hearing loss and hypertension in subjects. The participants were divided by gender. Orange bars represent low exposure subjects, and blue bars represent high exposure subjects. The gender is indicated on the left of each bar. An asterisk indicates that a significant effect (*P* < 0.05) was found for that gender.

We analyzed associations of health conditions and occupational noise level versus gender. In female workers, there was no association of the prevalence of hypertension or hearing loss with occupational noise exposure. Yet, significant differences were found in males between these two exposure levels. Figure 1 shows that in the male workers, the prevalence of hearing difficulty in the low exposure group was 22.13%, while in the high exposure group it was 26.33%, which was 1.2 higher, and statistically different from the low exposure group (*P* = 0.006). In addition, the risk of hypertension in high exposure group was worse than low exposure group (*Adjusted OR* = 1.25, *95% CI*, 1.01–1.54). These results indicate a distinct effect of high-level occupational noise on health conditions according to subjects’ gender.

### Stratified analysis between the two exposure groups by occupational duration to noise

After stratifying by duration time to occupational noise, we divided subjects into three categories (**<**3, 3–10, and **>**10 years). Exposure to high noise level demonstrated a significant association with hypertension and hearing loss when duration time to occupational noise was longer than 10 years (Table 4). In an analysis of the longest duration group (**>**10 years), the prevalence of hypertension showed a significant difference between the high exposure level and the low exposure level group (*P* = 0.010); the risk of hypertension in high group was about 1.69 times higher than in low group (*Adjusted OR* = 1.69, *95% CI*, 1.11–2.56). Similar results also occurred in the distribution of hearing loss, which showed that the incidence of hearing loss in high exposure group was statistically different from that in low exposure group (*P* < 0.000); the prevalence of hearing loss in high exposure group was 57.42%, which was approximately twice higher than in low exposure group (*Adjusted OR* = 3.60, *95% CI*, 2.51–5.16). However, in regard to a duration time of fewer than 10 years, there were no significant difference between the two exposure groups.

**Table 4:** Stratified analysis of cardiovascular and hearing conditions between the two exposure groups by occupational duration for noise.

Variable		High exposure levelv N (%)	Low exposure level^*c*^ N (%)	*P* value^*a*^	*Crude OR* (*95% CI*)	*Adjusted OR* (*95% CI*)^*b*^
**<3 years**						
Hypertension	Without	362 (90.05)	1434 (89.96)	1.000	0.99 (0.69–1.43)	0.99 (0.68–1.44)
	With	40 (9.95)	160 (10.04)			
Hearing Loss	Without	335 (83.33)	1293 (81.12)	0.349	0.86 (0.64–1.15)	0.85 (0.63–1.14)
	With	67 (16.67)	301 (18.88)			
**3–10 years**						
Hypertension	Without	421 (86.98)	1644 (89.30)	0.168	1.25 (0.92–1.69)	1.31 (0.97–1.79)
	With	63 (13.02)	197 (10.70)			
Hearing Loss	Without	372 (76.86)	1449 (78.71)	0.386	1.11 (0.88–1.41)	1.15 (0.90–1.47)
	With	112 (23.14)	392 (21.29)			
**>10 years**						
Hypertension	Without	116 (74.84)	612 (83.95)	**0.010**	**1.76** (**1.16–2.66)**	**1.69** (**1.11–2.56)**
	With	39 (25.16)	117 (16.05)			
Hearing Loss	Without	66 (42.58)	533 (73.11)	**0.000**	**3.67** (**2.56–5.25)**	**3.60** (**2.51–5.16)**
	With	89 (57.42)	196 (26.89)			

^a^Two-sided χ^2^ test for comparing the rate of hypertension, abnormal ECG, and hearing loss between the two groups.

^b^Adjusted for sex and occupational exposure time for noise in the logistic regression model.

^c^Reference group.

## Discussion

This study reported the prevalence of hypertension, abnormal ECG, and abnormal hearing thresholds in workers exposed to occupational noise in Nanjing city. Furthermore, we assessed the association between noise exposure and the incidence of hypertension, abnormal ECG, and hearing loss. We found that subjects exposed to intense ambient noise (high noise level) had significantly higher dangers of hypertension and hearing loss than subjects who were exposed to a lower noise level (*P* < 0.05). The risks of hypertension and hearing loss were elevated especially in male workers whose ages were between 30 and 45 years, or whose exposure times were longer than 10 years.

Noise as a kind of psychosocial stressor may cause hypertension through activating sympathetic nervous systems and the hypothalamus pituitary adrenal axis, which may motivate sequential elevated levels of adrenaline, noradrenaline, and cortisol^14–17^. Unfortunately, these three hormones also regulate blood pressure^18,19^. In addition, Ghotbi M. R. *et al*. had shown that the level of stress hormones, especially catecholamine significantly increased with noise exposure increment^15^. And some researchers had found that catestatin may inhibit the sympathetic activation in hypertension, and may involve in the pathogenesis of hypertension^20,21^. In regards to occupational noise levels above 85 dB(A), the association between noise exposure and hypertension is inconsistent. Some researchers have showed that occupational noise exposure is associated with a higher risk of hypertension or with a sustained elevation of blood pressure^1,2,22,23^, which is consistent with our findings (Table 2). Still, other studies have not suggested any significant link^11,24,25^. The difference between these results may be attributed to the different usage of personal protective equipment among different workers who exposed to high-frequency noise. Hence, we need to choose outer-ear measurements of noise levels alone as a source of exposure bias because they do not influence the true intensity of inner-ear exposure.

A number of epidemiological studies have demonstrated that high-frequency hearing loss is resulted from occupational noise exposure^26–30^. Chang suggests that, in aircraft-manufacturing workers, high-frequency hearing loss is a good biomarker for long occupational noise exposure^31^, which shows a trend similar to our research. Table 3 illustrates how a exposure of high-level occupational noise resulted in a significantly elevated risk of hearing loss especially for workers between the ages of 30 and 45. It should be emphasized that aging is the most common reason for sensorineural and noise-induced hearing abnormalities, and both related closely with the formation of ROS (reactive oxygen species). Researchers suggested that hearing damage is associated with the outer hair cell apoptosis pathway which due to oxidative stress involving oxidative damage to biological molecular (such as nucleic acids, lipids, proteins and so on) and accompanies progression of several physical conditions, including neurodegenerative, cardiovascular, eproductive and kidney diseases, and also different cancers^32^.

Participants were grouped by noise exposure duration and tested for associations in separate groups, we found that a significant association of hearing loss and hypertension with high-frequency noise in the group whose exposure time was longer than 10 years (Table 4). Also, in one industrial-based study, workers with abnormal audiograms had significantly longer noise exposure time relative to those with normal audiograms (*P* < 0.001)^33^. These results indicate that the risk of hearing loss or hypertension will be even worse when workers are exposed to higher occupational noise and for a longer duration. However, this is not unexpected because higher level of occupational noise and longer duration will be more dangerous, consequently, a more significant impact would be anticipated.

No significant association was measured between noise exposure levels and ECG conditions. This may be due to a lack of representative sample size, or other unknown environmental factors. Whatever the cause, whether high noise levels contribute to abnormal ECG is a question which remains unclarified.

There were several limitations to our study. A cross-sectional analysis showing a temporal problem might astrict the evidence for a causal relationship between occupational noise and unhealthy condition. However, the causal association between noise exposure and hearing abnormality has been acknowledged^34,35^. The variable of duration to occupation noise was based on own oral-report which could have led to bias, as workers may not have remembered clearly or could have potentially given false information about longer exposure time. Additionally, several potential confounders are known to cause hypertension or other cardiovascular diseases were not considered as covariates in our study, including a family history of hypertension, lack of exercise, and poor nutrition in daily diet^36–38^. Given that these are also risk factors for cardiovascular disorders, the true prevalence and association between noise exposure and health condition may be more significant than estimated in this study.

Protecting noise-exposed workers by reducing noise exposure in work places and improving safeguard procedures will be very important for preventing noise-induced hearing loss and other physiological abnormalities. Noise-exposed workers need to wear earplugs or earmuffs correctly. Worksite health programs including monitoring hypertension and other cardiovascular diseases should also focus on noise-exposed workers. Those who are identified in screening to have hypertension, hearing loss or abnormal ECG should be advised to change to a different work post which has less or no noise exposure.

Finally, although this study contributed several significant results towards the correlation between occupational noise and cardiovascular disorders, as well as hearing conditions, further human and animal studies are needed to explore the exact nature and basic mechanism for this association.
